# A Campaign of Education1Read before the joint-meeting of the Iowa State Veterinary Medical Association and the Iowa State Agricultural Society, January 10, 1899.

**Published:** 1899-03

**Authors:** J. M. Emmert

**Affiliations:** Atlantic, Iowa; Ex-President of the Iowa State Medical Society; Member of the American Medical Association; Member of the American Public Health Association; Ex-President Iowa State Board of Health


### A CAMPAIGN OF EDUCATION.[1]

By J. M. Emmert, M.D.,
ATLANTIC, IOWA.
EX-PRESIDENT OF THE IOWA STATE MEDICAL SOCIETY; MEMBER OF THE AMERICAN MEDICAL ASSOCIATION; MEMBER OF THE AMERICAN PUBLIC HEALTH ASSOCIATION; EX-PRESIDENT IOWA STATE BOARD OF HEALTH.

We have heard much the last few years of campaigns of education. The great political battle of 1896 was called a campaign of education, because almost every man, woman, and child in this great country was studying the money question, and the most learned men on both sides of the question were employed to prepare circulars, leaflets, newspaper and magazine articles, which were scattered abroad by the millions. The question of slavery was settled by force of arms, but only after the people had been aroused by the teachings of Wendell Phillips, Lloyd Garrison, and Horace Greeley. But, notwithstanding the newspaper campaign of these men, and the eloquent addresses in the halls of Congress, the one quiet, pathetic, earnest, and truthful appeal made by Harriet Beecher Stowe through *Uncle Tom's Cabin* was the small still voice that aroused the people to action, and slavery went down before its mighty influence. Thus it is that in all reforms it is the quiet but persistent work by those who are devoted to the cause that will win in the end.

I have selected this subject for your consideration to-night because I regard you as a band of educated and scientific men who are engaged in the great warfare against filth and disease, and preach the gospel of cleanliness and happiness, and I want to welcome you as co-workers in this field with the profession to which I have the honor to belong. The day of the "horse-doctor" is past and gone, and the educated veterinarian has taken his place, full of enthusiasm, zeal, and a desire to understand the underlying causes of disease, and if possible to remove them. He is satisfied with nothing but a scientific explanation of all questions pertaining to his profession. I am rejoiced to find so many of the profession not only interested in sanitary questions, but practical sanitarians.

[1] Read before the joint-meeting of the Iowa State Veterinary Medical Association and the Iowa State Agricultural Society, January 10, 1899.

As the people become educated to your worth, not only as veterinarians, but as sanitarians, your sphere of usefulness will be extended.

You are presumed to understand the diseases of animals both alive and dead, and able to point out and explain the difference between diseased and healthy food-products, especially meat and milk. This being the case, no man in the community can be of as much benefit upon the local boards of health as the learned veterinarian, and I think the time is not far distant, or at least I hope so, when every slaughter-house, meat-market, dairy, or other place where food-products are bought and sold, will be inspected under the supervision of the local boards of health. When this time does come, gentlemen, you will be called upon for your services, and the people will demand that they have the benefit of your knowledge. In fact, it is your duty, as well as mine, to assist in bringing about this happy result. You understand the dangers of eating meat infected by trichinosis and actinomycosis, or drinking milk infected by tubercle bacilli. Along with the medical profession and sanitarians, it is your privilege, as well as duty, to be teachers, leaders, and educators in this great work. The field is not only great, but the work is glorious.

No man can fill the niche in the world that God intended he should fill and live for himself alone. What we do to elevate and make humanity better, place our fellow-man upon a higher plane of usefulness, make him happier, wiser, and more prosperous, will alone secure the greatest happiness for ourselves, and make us feel that we have not lived in vain. You will be confronted by many difficulties and all kinds of obstructions to be overcome in your fight against ignorance, prejudice, sordid and avaricious desire to accumulate wealth. All these obstacles must be removed or overcome before the people will understand and appreciate the fact that they have an abiding interest in the fight against filth and disease. Your fight is in the interest of humanity, not of life alone, but the protection and preservation of property as well. I will not attempt to go into statistics to any extent to prove any assertion I may make; but suffice it to say that one-half of the deaths are caused by contagious diseases, all of which are preventable diseases, and should be eradicated, and would be if proper sanitary laws were upon our statute-books and heroically enforced.

The existence and spread of typhoid fever, scarlet fever, diphtheria, tuberculosis, hog-cholera, and actinomycosis are *prima facie* evidence of either our ignorance or criminal negligence, and an insult to our higher civilization. The loss of life and destruction of property by these preventable diseases are beyond our conception unless studied in the light of modern scientific statistics. Take as an illustration tuberculosis, or the great "white plague," as it is sometimes called. One out of every seven deaths throughout the world is caused by tuberculosis. Iowa contributes nine deaths every day, and three thousand every year; the United States contributes one hundred and fifty thousand, and the world five millions per year. Contemplate these figures for a moment, then ask yourself why the people are not aroused to the dangers that surround them, and demand that our lawmakers, both State and National, shall enact laws to stamp out this disease, or at least prevent its fearful ravages. If a case of smallpox is reported in this or adjoining States, the State board of health at once makes preparations to prevent its spread. Newspapers take up the cry of danger, and the people become excited and demand that the local boards of health be doubly vigilant.

Last winter the House of Representatives of this State sent Dr. Kennedy to the northern part of the State to investigate a case of leprosy, and see what was necessary to protect the State from its ravages. What a farce! This is literally straining at a gnat and swallowing a camel. More people die in Iowa of tuberculosis in one day than ever did or will die of leprosy. And more people die of tuberculosis in one month than have died of smallpox in the last ten years. The almost complete stamping out of smallpox is a beautiful result and a scientific explanation of what could be done by preventive medicine, and to-day if the laws throughout the united world compelling the vaccination of every child at a certain age were enforced, smallpox would in ten years die, a lost disease.

Now, in my judgment, tuberculosis is just as easily stamped out as smallpox. It may take generations to do it, but proper legislation, and its complete enforcement throughout the world—I mean a concerted action by all the powers of the world—will reduce the mortality due to the disease to a minimum, if not entirely wipe it out.

But to do this we must start right; we must understand thoroughly the etiology of the disease, its natural history, how it spreads, etc. Every case of tuberculosis comes from some other case of tuberculosis in either man or beast. There is no such thing as spontaneous generation in tuberculosis; the germs causing the disease do not arise that way. Every germ must come from a preexisting germ, and grown in a suitable medium for the disease; but if they never get any germs into their system they will never have tuberculosis. Then what must we do to prevent their taking the disease? Why, of course, keep them away from the disease, and in this way keep the disease away from them. A large percentage of the cases are caused by the inhalation of germs floating in the air, and which were thrown off by expectoration from the human being, discharges from the nostrils of animals, or from the bowels of both. These discharges are dried and float in the air, and are drawn into the lungs; or, as in animals, they are rubbed on troughs or other material, to be licked up by the animal.

But this is not the only way the disease can be contracted. Tuberculous meat and milk are responsible for a certain percentage of cases. Meat if properly cooked can be eaten with impunity; but we do not yet understand what is properly cooked tuberculous meat. A certain temperature may kill the bacilli, but not the spores. So the safest way is not to eat it at all. In my judgment a large majority of the bottle-fed children reported as having died of marasmus, cholera infantum, and other wasting diseases, the cause of death was intestinal or meningeal tuberculosis from tuberculous milk.

But there is another and very important side to this question, and one which our agricultural friends are especially interested in: The loss of cattle by this disease alone runs into the millions of dollars. The property value of stock— those diseased—is hard to estimate, as no statistics, to my knowledge, has ever been gathered. I know one stockraiser in my part of the State who had destroyed, by our efficient State veterinarian, for tuberculosis, five thousand dollars' worth of high-bred cattle. This gentleman had a practical but very costly lesson, and he intends to profit by it, as he wrote me in answer to an inquiry, that in the future every animal leaving his yard for breeding purposes will carry with it a certificate showing that it had been tested by tuberculin, and was free from tuberculosis; he also said that every animal coming on his place must have the same kind of certificate. I do not want to see every farm in Iowa take the same lesson, but I do hope they will profit by this gentleman's experience.

But there is still another side to this question that touches every farmer and dairyman. Iowa is fast becoming the greatest dairy State in the Union. I find in the *Tenth Annual Report of the State Dairy Commission* that there were shipped out of the State 80,032,916 pounds of butter, an increase in one year of 13,535,808 pounds, and 626,632 pounds of cheese. This only represents a certain percentage of the product manufactured, and does not include the raw milk used as food, buttermilk, skimmed milk, etc.; but you have some idea of the immensity of the dairy interest from these figures. Now we must have a market for this product, and an increasing and steady market. Our very efficient Secretary of Agriculture recognized this fact, and upon taking office proceeded at once to introduce American butter into the Liverpool and other English markets. These markets have been mainly supplied by the dairy interests of Northern Europe, where tuberculosis exists as well as here. I believe it is an acknowledged fact that butter made from tuberculous milk will contain the bacilli. This being the case, as long as we export butter containing these germs we cannot expect to excel in the markets of the world; but stamp out tuberculosis in Iowa, and then stamp on every case of butter that goes out of the State, "Guaranteed free from pathogenic germs;" make the word "Iowa" upon every case stand for "Purity," and the demand will soon be so great that the dairy interest of the State will be doubled, and Iowa will at once step to the front as the greatest dairy section in the world.

What I have said about tuberculosis from a money stand-point can be applied to hog-cholera; hog-cholera is a contagious disease, and consequently can be prevented and finally stamped out if the proper remedy is applied. You at once ask me for the remedy. This every sanitarian and scientific student understands. It can be summed up in one word—"segregation." Every animal when once infected should be destroyed. And when I say destroyed, I do not mean killed and used as human or animal food, allowed to lay and rot, or even buried; but burned, cremated, disinfected by fire. The township trustees and city and corporation councils, as boards of health, led by an intelligent veterinarian, should have charge of all suspicious animals, and not only order their destruction and final cremation, but do it themselves, or have an officer delegated for this work.

But it is not sufficient to destroy our diseased stock; we must have some means by which we can keep diseased stock out of the State. You cannot stop a stream by damming it up—stop the source and the stream will dry up. So it is with tuberculosis and other contagious diseases among stock: destroy the disease at home, and keep outside diseased stock from coming in, and our herds will soon be free from contagious disease. The cattle-men and hog-men tell me this kind of legislation would ruin their business. You will find that this argument is used only by the middlemen, the men who buy and ship, and care little whether the stock handled is healthy or diseased; but take the farmersand stock raisers, who take pride in their business, and who are interested in the growth and development of our beautiful State, and I think you will find them almost to a man interested in this subject, and willing and anxious to do everything possible to stamp out contagious diseases, and thereby give protection to the people and property of the State.

Now, gentlemen, the people must be educated along these lines, and when they once understand that quarantine means protection to life and property, they will not only demand that none but healthy animals enter the State, but that all diseased animals in the State shall be destroyed. Should this be accomplished a wonderful impetus will be given to the stock, meat, and dairy interests, and Iowa with one bound will leap to the forefront as the greatest stock and dairy country in the world.

